# A retrospect study based on real-world data to observe metabolic function in cancer patients using albumin-bound paclitaxel

**DOI:** 10.1038/s41598-023-35992-x

**Published:** 2023-06-03

**Authors:** Qi Zheng, Hanzhou Wang, Chao Xue, Shuhan Yang, Yan Wang, Wei Hou, Ying Zhang

**Affiliations:** 1 Oncology Department, Guang’anmen Hospital, China Academy of Chinese Medical Sciences, Beijing, 100053 China; 2 Pneumology Department, Guang’anmen Hospital, China Academy of Chinese Medical Sciences, Beijing, 100053 China

**Keywords:** Medical research, Molecular medicine, Oncology

## Abstract

There is substantial evidence that albumin-bound paclitaxel (nab-paclitaxel) is effective and safe for the treatment of breast, lung and pancreatic cancers. However, it can still cause adverse effects by affecting cardiac enzymes, hepatic enzyme metabolism and blood routine related indicators, which affects the use of chemotherapy for a full course of treatment. However, there are no relevant clinical studies to systematically observe the effects and dynamics of albumin-bound paclitaxel on cardiac enzymes, liver enzyme metabolism, and routine blood-related indices. The purpose of our study was to determine the levels of serum creatinine (Cre), aspartate aminotransferase (AST), alanine aminotransferase (ALT), lactate dehydrogenase (LDH), creatine kinase (CK), creatine kinase isoenzyme (CK-MB), white blood cells (WBC) and hemoglobin (HGB) in cancer patients treated with albumin-conjugated paclitaxel. This study retrospectively analyzed 113 patients with cancer. Patients who had received two cycles of nab-paclitaxel 260 mg/m^2^ (administered intravenously on days 1, 8, and 15 of each 28-day cycle) were selected. Serum Cre, AST, ALT, LDH, CK, and CK-MB activities, WBC counts, and HGB levels were measured before and after treatment with two cycles. Fourteen cancer types were analyzed. The distribution of cancer types in patients was mainly concentrated in lung, ovarian, and breast cancer. Nab-paclitaxel treatment markedly decreased Cre, AST, LDH, and CK activities in the serum and WBC counts and HGB levels, respectively. Serum Cre and CK activities and HGB levels were remarkably downregulated at baseline compared to healthy controls. Patients receiving nab-paclitaxel treatment cause metabolic disorders in tumor patients by reducing the decrease of Cre, AST, LDH, CK, CK-MB, WBC and HGB indexes, thus inducing the occurrence of cardiovascular events, hepatotoxic events and fatigue and other symptoms. Therefore, for tumor patients, although receiving nab-paclitaxel improves the anti-tumor effect, it is still necessary to closely monitor the changes of related enzymatic and routine blood indicators, so as to detect and intervene at an early stage.

## Introduction

Paclitaxel is widely used and plays an important role in the chemotherapy of breast cancer, lung cancer, pancreatic cancer, and other cancers, which is a typical pharmaceutic preparation of natural anti-cancer drugs found in plants. Albumin-bound paclitaxel (nab-paclitaxel) is a solvent-free albumin-bound form of paclitaxel, which not only solves the allergy problem of paclitaxel but also improves its therapeutic effect. In clinical studies, nab-paclitaxel has been found to have activity against various advanced solid tumors, including those of the breast, lungs, and stomach. In the treatment of breast cancer, conventional paclitaxel therapy can only achieve a 19% remission rate, whereas nab-paclitaxel can improve the remission rate to 33%^1^. For non-small cell lung cancer (NSCLC), the objective response rate was 29.9% for nab-paclitaxel compared with 15.4% for docetaxel^2^. The effect of albumin-paclitaxel is increased because the nanocarriers deliver the drug quickly to cancer tissue and stay there longer. However, only a few studies have focused on metabolic dysfunction. Unfortunately, metabolic dysfunction is a potential limitation associated with the long-term use of nab-paclitaxel in cancer, and requires further study to assess the value of nab-paclitaxel. Although nab-paclitaxel, an innovative form of paclitaxel, has superior antitumor effects. However, attention should also be paid to the adverse events it causes. A meta-analysis including 12 clinical trials reported that grade 3/4 anemia, thrombocytopenia, and neurotoxicity were more common with nab-paclitaxel than with conventional paclitaxel^3,4^. The relative risk of nab-paclitaxel compared to paclitaxel were not increased for all-grade and high-grade peripheral neuropathy^5^. In addition, paclitaxel has been reported to cause bradycardia by affecting myocardial metabolism, with an incidence of 30%^6^, and to affect liver enzyme metabolism^7^. Therefore, during the use of albumin, blood tests, liver enzymes and cardiac enzymes should be closely monitored for early detection of adverse effects and timely intervention.

Alanine aminotransferase (ALT) and aspartate aminotransferase (AST) are liver function enzymes that have been shown to reflect disease severity in several chronic liver diseases and are widely distributed in a variety of cells, most notably liver cells. ALT and AST play key roles in amino acid metabolism, purine/pyrimidine base synthesis, urea and protein synthesis, and gluconeogenesis^8^. Creatinine (Cre) is a product of muscle metabolism in the human body and is excreted mainly by glomerular filtration. Lactic dehydrogenase (LDH) is an enzyme that plays an important role in body energy metabolism. It can be found in almost all tissues, including the blood, heart, kidneys, brain, and lungs. LDH is released from damaged tissues and can serve as a biomarker of damaged heart tissue. Creatine kinase (CK) is a guanidino-kinase that catalyzes the reversible phosphorylation of creatine to phosphocreatine, and is primarily distributed in the bone and myocardium. The plasma activity of creatine kinase isoenzyme (CK-MB), an isoenzyme of CK, is generally used to evaluate acute coronary syndromes. The detection of serum CK isozymes and CK-MB is helpful in determining the degree of myocardial metabolism. Comprehensively, monitoring serum ALT, AST, Cre, LDH, CK, and CK-MB activities for liver, renal, muscle, and cardiac biomarkers can be valuable for assessing systemic metabolism in patients^9,10^.

Nab-paclitaxel is efficacious and safe for the treatment of solid tumors. Unfortunately, few studies have focused on measuring the changes in serum ALT, AST, Cre, LDH, CK, and CK-MB activities during nab-paclitaxel treatment. In this study, we conducted a retrospective investigation focused on measuring the changes in serum ALT, AST, Cre, LDH, CK, and CK-MB levels in cancer patients receiving nab-paclitaxel targeted therapy. White blood cell (WBC) and hemoglobin (HGB) levels were also observed. The results detected that in serum ALT, AST, Cre, LDH, CK activities, WBC counts and HGB levels of patients who had used nab-paclitaxel were likely to have descended.

## Materials and methods

### Patients

From January 2018 to December 2021, cancer 113 patients who had used two cycles of nab-paclitaxel (260 mg/m^2^) administered intravenously on days 1, 8, and 15 of each 28-day cycle were selected. Patients treated with nab-paclitaxel at the Oncology Department, Guang’anmen Hospital, China Academy of Chinese Medical Sciences were retrospectively recruited for this study. Patients with active infections, systemic corticosteroid therapy within 1 year, or hematological malignancies that might affect the test criteria were excluded. We included 113 healthy controls from the health examination Center of Guang 'anmen Hospital, China Academy of Chinese Medical Sciences. These individuals were matched to the gender and age of the patients included above.

### Data collection

Variables such as age, sex, histological diagnosis, and smoking and alcohol use history were extracted from medical records. The first effective evaluation was performed prior to treatment. Follow-up evaluation was performed after the first and second cycles (administered intravenously on days 1, 8, and 15 of each 28-day cycle) of treatment. Additionally, routine complete blood counts (WBC and HGB levels), biochemical tests (including Cre, AST, ALT, and LDH activities), and coagulograms (CK and CK-MB activities) were performed. The laboratory of Guang ‘anmen Hospital of the Chinese Academy of Medical Sciences tests and issues all hematological results. The instruments used are automatic hematology analyzer (Sysmex XN-10™) and automatic chemistry analyzer (AU5800 series, Beckman Coulter).

### Statistical analysis

In the statistical analysis of the research data, GraphPad Prism8 (GraphPad Software, San Diego, CA, USA) and SPSS statistical software version 24.0 (SPSS Inc., Chicago, IL, USA) were used. Considering the predictor variables, such as age, sex, histological diagnosis, smoking, and drinking history, statistics were obtained using mixed linear modeling. A normality test was initially performed. Normally distributed data are expressed as mean and standard deviation (SD). The differences between the patients at baseline and after the first and second treatment groups and the matched healthy control group were compared using the paired t-test. The differences between the patients at baseline and the matched healthy control group were compared using the independent-samples t test. If the data were not normally distributed, the median, interquartile range, and min–max. The differences between patients at baseline and after the first and second treatment groups and the matched healthy control group were compared using the Wilcoxon matched-pairs signed rank test. The differences between patients at baseline and the matched healthy control group were compared using the Mann–Whitney test. Statistical significance was defined as a two-sided *P*-value < 0.05.

### Ethics approval and consent to participate

The study protocol was approved by the Ethics Committee of Guang’anmen Hospital, China Academy of Chinese Medical Sciences. Due to the retrospective nature of the study, the informed consent was waived by the Ethics Committee of Guang’anmen Hospital, China Academy of Chinese Medical Sciences. However, we guaranteed this opportunity by opt-out. All procedures performed in this study involving human participants were in accordance with the ethical standards of the national research committee and with the 1964 Helsinki Declaration and its later amendments or comparable ethical standards. Our team acquired administrative permission to access the data used in this study.

## Results

### Patient characteristics

A total of 113 patients were treated with nap-paclitaxel during the study period. Table 1 lists the detailed characteristics of the patients. There were 43 (38.1%, 95% CI: 0.291–0.470) men and 70 (61.9%, 95% CI: 0.530–0.709) women in the total cohort, with a median age of 61 years (Quartiles 25–75%, 55–69 years). Among the patients, 29.2% (33/113, 0.208–0.376) had a smoking history and 22.1% (25/113, 0.145–0.298) had a drinking history. Meanwhile, a total of 36.3% (41/113, 95% CI: 0.274–0.451) patients had lung cancer, 16.8% (19/113, 95% CI: 0.099–0.237) had ovarian cancer, 14.1% (16/113, 95% CI: 0.077–0.206) had breast cancer, 6.2% (7/113, 95% CI: 0.018–0.106) had cervical cancer, 6.2% (7/113, 95% CI: 0.018–0.106) had esophagus cancer, 5.3% (6/113, 95% CI: 0.012 ~ 0.094) had pancreatic cancer and 4.4% (5/113, 95% CI: 0.006–0.082) had gastric cancer. Small numbers of endometrial cancer (2.7%, 3/113), endometrial cancer (2.7%, 3/113), nasopharyngeal cancer (1.8%, 2/113), peritoneal malignancy (0.9%, 1/113), urethral carcinoma (0.9%, 1/113), gallbladder carcinoma (0.9%, 1/113), and thymoma (0.9%, 1/113) cases were also included. A large proportion of patients (83.2%, 94/113) were at stage IV. Table 1.

**Table 1:** Patients’ characteristics.

Variables	N	%	95% CI
Age			
Median	61		
Quartiles 25–75%	55–69		
Gender			
Male	43	38.1	0.291–0.470
Female	70	61.9	0.530–0.709
Smoking history			
Yes	33	29.2	0.208–0.376
No	80	70.8	0.624–0.792
Drinking history			
Yes	25	22.1	0.145–0.298
No	88	77.9	0.702–0.855
Cancer type			
Lung cancer	41	36.3	0.274–0.451
Ovarian cancer	19	16.8	0.099–0.237
Breast cancer	16	14.1	0.077–0.206
Cervical cancer	7	6.2	0.018–0.106
Esophagus cancer	7	6.2	0.018–0.106
Pancreatic cancer	6	5.3	0.012–0.094
Gastric cancer	5	4.4	0.006–0.082
Endometrial cancer	3	2.7	− 0.003–0.056
Endometrial cancer	3	2.7	− 0.003–0.056
Nasopharynx cancer	2	1.8	− 0.007–0.042
Peritoneal malignancy	1	0.9	− 0.008–0.026
Urethral carcinoma	1	0.9	− 0.008–0.026
Gallbladder carcinoma	1	0.9	− 0.008–0.026
Thymoma	1	0.9	− 0.008–0.026
Tumor stage			
I	6	5.3	0.012–0.094
III	13	11.5	0.056–0.174
IV	94	83.2	0.763–0.901

### Evaluation of serum Cre, AST, ALT, LDH, CK and CK-MB activities and WBC, HGB levels in patients and health control

**Figure 1:**
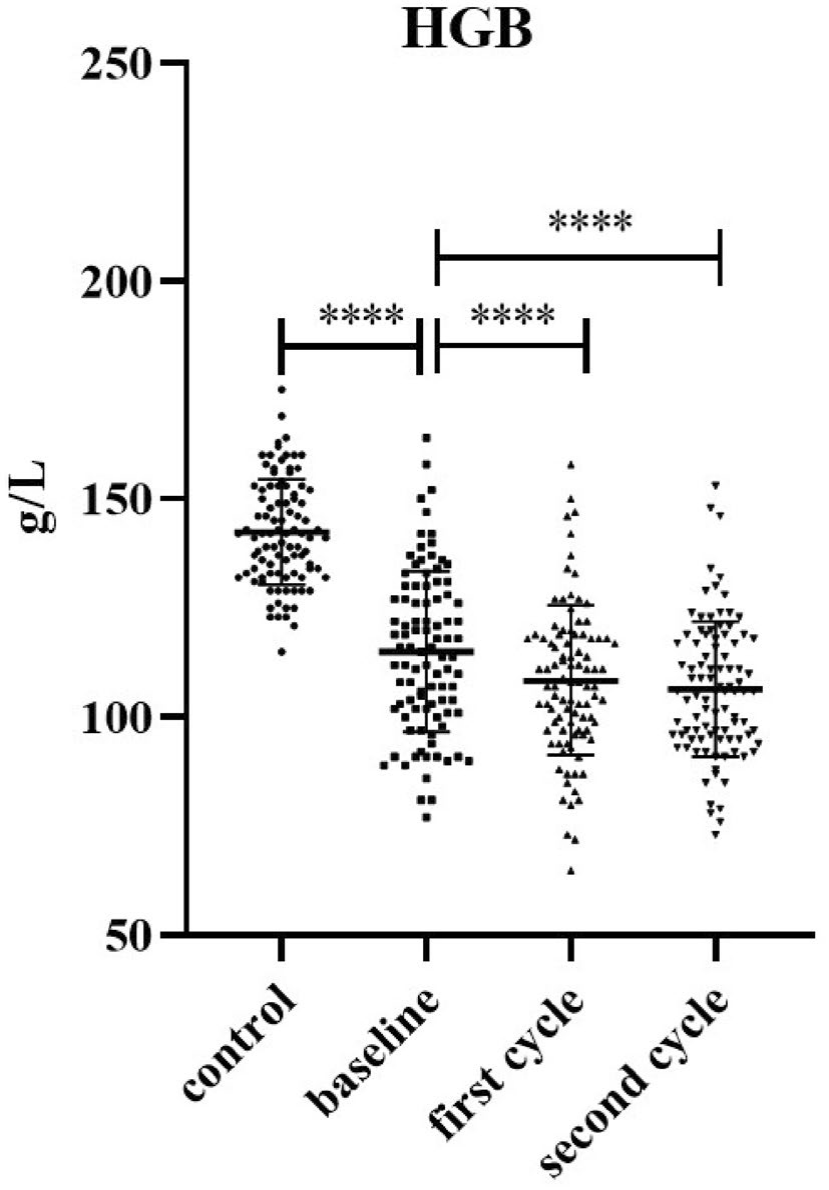
HGB levels of in patients and health control. **** *P* < 0.0001.

Serum activity levels of Cre, CK, CK-MB, AST, ALT and LDH in patients and healthy controls are shown in Table 2, as well as detailed characteristics of WBC and HGB levels. Healthy controls were matched for serum Cre, CK, CK-MB, AST, ALT and LDH levels, besides WBC and HGB levels. HGB levels were markedly decreased in patients at baseline compared with healthy controls (*P* < 0.0001) (Fig. 1, Table 2). In addition, serum Cre, CK, and ALT levels were significantly lower in patients at baseline than in healthy controls (*P* < 0.0001, *P* < 0.05) (Table 2).

**Table 2:** The detailed characteristics of serum Cre, AST, ALT, LDH, CK and CK-MB activities and WBC levels of in patients and health control.

	Group	Number	Median	Interquartile range	Min	Max	*P* value
WBC	Baseline	96	5.95	2.77	1.76	18.07	
	First cycle	96	4.67	2.20	1.79	18.61	< 0.0001
	Second cycle	96	4.80	2.40	1.17	13.71	< 0.0001
	Health Control	96	6.33	1.68	3.57	9.07	0.1303
Cre	Baseline	110	54.50	20.50	30.00	97.00	
	First cycle	110	52.00	19.00	30.00	92.00	< 0.0001
	Second cycle	110	52.00	17.00	25.00	97.00	< 0.0001
	Health Control	110	66.00	16.25	45.00	101.00	< 0.0001
AST	Baseline	113	19.00	12.20	8.00	99.70	
	First cycle	113	19.10	9.35	10.40	71.10	0.0122*
	Second cycle	113	19.40	9.05	10.60	125.00	0.1034
	Health Control	113	20.60	7.10	13.80	33.80	0.3435
ALT	Baseline	111	15.60	14.30	3.20	77.80	
	First cycle	111	15.60	12.00	2.80	71.50	0.3929
	Second cycle	111	13.50	10.70	3.30	86.30	0.4693
	Health Control	111	16.60	9.20	7.70	46.20	0.0393*
LDH	Baseline	106	174.00	92.00	108.00	787.00	
	First cycle	106	178.50	56.75	96.00	489.00	0.0307*
	Second cycle	106	171.00	52.00	96.00	731.00	0.0052**
	Health Control	106	166.00	33.75	118.00	250.00	0.0160*
CK	Baseline	98	46.00	33.50	14.00	171.00	
	First cycle	98	40.00	30.00	13.00	225.00	0.0249*
	Second cycle	98	43.00	29.25	14.00	222.00	0.0700
	Health Control	98	94.50	48.50	37.00	171.00	< 0.0001
CK-MB	Baseline	102	10.00	6.25	1.00	72.00	
	First cycle	102	10.00	5.00	3.00	42.00	0.0805
	Second cycle	102	9.00	6.00	3.00	63.00	0.1964
	Health control	102	11.00	3.00	5.00	24.00	0.2759

In our laboratory, 3.5–9.5 × 10^9^/L is adopted as the cut-off value for normal WBC; 115–150 U/L is adopted as the cut-off value for normal HGB; 45–84 μmol/L is adopted as the cut-off value for normal Cre; 13–35 U/L is adopted as the cut-off value for normal AST; 7–40 U/L is adopted as the cut-off value for normal ALT, < 247 U/L is adopted as the cut-off value for normal LDH, < 145 U/L is adopted as the cut-off value for normal CK, and < 25 U/L is used as the cut-off value for normal CK-MB.

### The effects of predictor variables on Cre, AST, ALT, LDH, CK and CK-MB activities and WBC, HGB levels in patients

Considering the predictor variables, such as age, sex, histological diagnosis, smoking, and drinking history, statistics were performed using mixed linear modeling. As Table 3 shows, the influence of age, sex, cancer type, stage, smoking, and drinking history were eliminated in this study.

**Table 3:** Type III test of fixed effects of WBC, HGB, AST, ALT, LDH, CK and CK-MB.

	Source	Numerator df	Denominator df	F	Sig
WBC	Age	1	68	13.320	0.001
	Gender	1	68.000	1.623	0.207
	Cancer type	14	68.000	1.655	0.087
	Stage	9	68.000	1.450	0.185
	Smoking history	1	68.000	0.220	0.641
	Drinking history	1	68.000	0.328	0.569
	Before/after treatment	2	190	12.229	0.000
HGB	Age	1	67.000	0.012	0.913
	Gender	1	67	0.043	0.836
	Cancer type	14	67	1.183	0.309
	Stage	9	67	1.241	0.286
	Smoking history	1	67	0.028	0.869
	Drinking history	1	67.000	0.039	0.845
	Before/after treatment	2	188.000	22.173	0.000
Cre	Age	1	83.000	0.915	0.342
	Gender	1	83	2.296	0.134
	Cancer type	14	83	1.566	0.106
	Stage	9	83	0.745	0.666
	Smoking history	1	83	0.000	0.989
	Drinking history	1	83	1.555	0.216
	Before/after treatment	2	220	17.046	0.000
AST	Age	1	79.454	0.826	0.366
	Gender	1	79.450	0.164	0.687
	Cancer type	13	83.207	2.028	0.028
	Stage	9	79.779	0.449	0.904
	Smoking history	1	79.791	0.443	0.508
	Drinking history	1	81.582	0.603	0.440
	Before/after treatment	2	218.008	2.173	0.116
ALT	Age	1	83.000	13.915	0.000
	Gender	1	83	0.214	0.645
	Cancer type	14	83	1.534	0.117
	Stage	9	83	1.362	0.219
	Smoking history	1	83	0.341	0.561
	Drinking history	1	83	3.367	0.070
	Before/after treatment	2	220.000	1.181	0.309
LDH	Age	1	79	0.365	0.548
	Gender	1	79	2.583	0.112
	Cancer type	14	79	5.160	0.000
	Stage	8	79	1.283	0.265
	Smoking history	1	79	0.324	0.571
	Drinking history	1	79	0.010	0.920
	Before/after treatment	2	210.000	9.878	0.000
CK	Age	1	72.000	0.002	0.967
	Gender	1	72.000	1.129	0.292
	Cancer type	13	72.000	0.400	0.966
	Stage	8	72.000	0.732	0.663
	Smoking history	1	72	0.043	0.835
	Drinking history	1	72	0.066	0.798
	Before/after treatment	2	194	2.182	0.116
CK-MB	Age	1	75.000	0.410	0.524
	Gender	1	75	0.451	0.504
	Cancer type	14	75.000	1.219	0.280
	Stage	8	75	0.138	0.997
	Smoking history	1	75.000	0.326	0.570
	Drinking history	1	75	1.477	0.228
	Before/after treatment	2	202	2.184	0.115

The influence of the above factors being excluded, the median WBC levels were 5.95 (2.77, 1.76–18.07) at baseline and 4.67 (2.20, 1.79–18.61), 4.80 (2.40, 1.17–13.71) after the first and second cycles of nab-paclitaxel treatment, respectively, which was significantly reduced (*P* < 0.001). The mean WBC levels were 21.51% and 19.32% higher at baseline than after the first and second cycles of nab-paclitaxel treatment, respectively (Table 2). The HGB data were normally distributed, as shown in Fig. 1. Compared to baseline, HGB levels significantly decreased after the first and second cycles of nab-paclitaxel treatment (*P* < 0.0001). Meanwhile, the median of Cre activities was 54.50 (20.50, 30.00–97.00) on baseline and 52.00 (19.00, 30.00–92.00), 52.00 (17.00, 25.00–97.00) after the first and second cycle nab-paclitaxel treatment, respectively, which was significantly reduced (*P* < 0.0001). The median LDH activities was 174.00 (92.00, 108.00–787.00) at baseline and 171.00 (52.00, 96.00–731.00) after the second cycle of nab-paclitaxel treatment, which was significantly decreased (*P* < 0.01). Similarly, the median CK activities was 46.00 (33.50, 14.00–171.00) at baseline and 40.00 (30.00, 13.00–225.00) after the first cycle of nab-paclitaxel treatment, which was markedly decreased (*P* < 0.05) (Table 2).

## Discussion

Nab-paclitaxel is a novel drug that binds paclitaxel to an albumin at the nanoscale. The use of human albumin as a delivery vehicle allows for direct administration without pretreatment with precorticosteroids and antihistamines, reduces allergic reactions, greatly increases bioavailability and dosage, and improves clinical efficacy. Although with great effectiveness and fewer allergic reactions, it has the side effects, mainly involve cardiovascular, respiratory, digestive, nervous, and blood systems.

In this real-world study, liver function, kidney, muscle and heart biomarkers, including Cre, AST, LDH and CK activities and WBC and HGB levels, were reduced during nab-paclitaxel treatment, suggesting that nab-paclitaxel use may be associated with an increased risk of metabolic disorders. The increase and decrease of these markers in this study did not exceed the upper and lower limits of normal, but may indicate a trend of accumulated fatigue and metabolic dysfunction with long-term use. The increase or decrease in the WBC count was mainly influenced by the number of neutrophils. Large changes in lymphocyte counts can also cause changes in WBC counts. The main reason for the decrease in HGB is the reduction in erythropoiesis, which can be caused by a variety of factors, including hematopoietic stem cell injury. The production and regulation of WBC originates from bone marrow hematopoietic stem cells. Similarly, all cell types with myeloid potential produce red blood cells (RBC), the predominant cell type produced by multipotent hematopoietic progenitors^11^. This study demonstrated that the level of HGB at baseline rapidly declined compared to that in matched healthy controls, which may be caused by decreased bone marrow metabolism. In addition, studies have shown that fatigue and neutropenia are frequent adverse reactions during nab-paclitaxel use, which may also be responsible for the reduction in WBC and HGB levels.

Cre is a metabolite of creatine, which is the main marker of renal function^12^. Elevated serum Cre activity is associated with an increased risk of kidney injury, morbidity, and mortality^13^. In contrast, a relatively low level of serum Cre is appreciated far less in clinical practice. A recent study showed that decreased Cre activity is an important risk factor for poor prognosis in patients in the intensive care unit^14^. CK is an important energy regulating enzyme in myocardium, and mainly exists in skeletal muscle, myocardium and brain tissue. CK disturbances of the CK were associated with various diseases, including cancer. In addition, Cre and Cre analogs such as cyclocreatine were found to have antitumor effects^12^. Interestingly, we detected that the common feature of serum Cre and CK activities notably declined in patients’ baseline, compared to matched healthy controls, which seems to be associated with decreased metabolic levels in patients. The serum Cre and CK activities should be focused on; CK along with its substrate Cre may be related to the growth of solid tumors and possibly their metastasis^15^.

AST and ALT are enzymes that help identify toxins in the liver, liver disease, and liver damage. As one of the most important transaminases in human body, AST exists in various tissues such as liver, myocardium, brain, skeletal muscle, pancreas, kidney and lung. AST is released into the bloodstream when tissue is damaged. An increase in serum AST activity is a sign of tissue injury; simultaneously, AST, as an indispensable evaluation index, is used in clinical evaluation of tumor drugs. ALT is mainly found in the liver, which is a sign of liver damage. High levels of serum AST and ALT have been shown to be markers of poor prognosis and favorable outcomes in pancreatic cancer, respectively^16,17^. Although serum elevation of AST and ALT did not exceed the upper limit of normal in patients in this study, serum ALT activity was significantly reduced compared with matched healthy controls. Therefore, clinicians should pay attention not only to the elevation of AST and ALT, but also to the downregulation of AST and ALT levels when reassessing a patient’s condition.

LDH plays an important role in cancer metabolism as an oxidoreductase that regulates the conversion of pyruvate to lactic acid during anaerobic glycolysis^18^. LDH is widely found in the cytoplasm and mitochondria of liver, heart, skeletal muscle, lung, spleen and other tissue cells. Elevated serum LDH level in patients with pancreatic cancer has been considered as a marker of tumor invasion, metastasis and poor prognosis^19^. Induced by hypoxia in the tumor microenvironment, cancer cells produce large amounts of lactic acid through glucose and glutamine metabolism^20,21^, and serum LDH levels are significantly elevated under hypoxic conditions^22^. Furthermore, serum LDH levels are associated with the systemic inflammatory response^19^. LDH can increase the production of reactive oxygen species. It also regulates apoptosis and autophagy^23^. In addition, aberrant metabolism, such as LDH levels out of the normal range, has been correlated with tumor aggressiveness and poor prognosis^24,25^. This study observed that serum LDH activities were significantly increased on the first cycle and decreased on the second cycle in the use of nab-paclitaxel. Serum LDH activity was higher in patients than in matched healthy controls. Therefore, the role of LDH in tumor biology is complex, and playing a greater role in the influence of tumor microenvironment may be a potential target for cancer treatment, and the mechanism of action needs to be further studied.

In addition, clinical observation has found that nab-paclitaxel is easy to cause fatigue and other uncomfortable symptoms in tumor patients. However, there is no targeted treatment for fatigue symptoms in tumor patients, and it is urgent to understand the mechanism of fatigue caused by nab-paclitaxel. Previous studies found that patients with tumor-related fatigue showed a downward trend in metabolic related indexes^26^. Based on the results of this study, we also found that albumin-binding paclitaxel could cause a decrease in cardiac, liver and kidney metabolism and blood routine related indexes. Consequently, routine blood and biochemical tests are necessary. Whether it is the mechanism causing fatigue in tumor patients still needs further research to confirm.

## Conclusions

The results of our study showed that patients who had used nab-paclitaxel showed a decreasing trend in serum Cre, AST, LDH, CK, WBC count and HGB levels, thus causing an increased probability of cardiovascular events, hepatotoxic events and fatigue and other symptoms. Therefore, when using nab-paclitaxel, changes in the above serological indicators should be monitored at all times to detect abnormalities early and intervene early to ensure that tumor patients receive a full course of chemotherapy, improve the effectiveness of antitumor therapy and improve the quality of prognosis survival. It should not be considered that the clinical application of albumin-conjugated paclitaxel is safe while improving the antitumor therapeutic effect of traditional paclitaxel after using modern technology. Rather, attention should be paid to the effect of drug toxicity on efficacy during practical clinical application.

However, the present study still has a small number of cases insufficient to support subgroup analysis, thus failing to clarify the differential effects of albumin-bound paclitaxel in different cancer types and the variability of occurrence of adverse events, and there is a need to expand the sample size to further corroborate the results of this study and reduce the bias of the results, so as to better guide clinical practice.

## Data Availability

Data used to support the findings of this study are available from the corresponding author upon request.

## References

[1] Gradishar WJ, Tjulandin S, Davidson N, Shaw H, Desai N, Bhar P (2005). Phase III trial of nanoparticle albumin-bound paclitaxel compared with polyethylated castor oil-based paclitaxel in women with breast cancer. J. Clin. Oncol..

[2] Yoneshima Y, Morita S, Ando M, Nakamura A, Iwasawa S, Yoshioka H (2021). Phase 3 trial comparing nanoparticle albumin-bound paclitaxel with docetaxel for previously treated advanced NSCLC. J. Thorac. Oncol..

[3] He F, Liu J, Shen X, Wang Z, Li Q, Li G (2022). Adverse event profile for nanoparticle albumin-bound paclitaxel compared with solvent-based taxanes in solid-organ tumors: A systematic review and meta-analysis of randomized clinical trials. Ann. Pharmacother..

[4] Tan H, Hu J, Liu S (2019). Efficacy and safety of nanoparticle albumin-bound paclitaxel in non-small cell lung cancer: A systematic review and meta-analysis. Artif. Cells Nanomed. Biotechnol..

[5] Peng L, Bu Z, Ye X, Zhou Y, Zhao Q (2017). Incidence and risk of peripheral neuropathy with nab-paclitaxel in patients with cancer: A meta-analysis. Eur J Cancer Care (Engl).

[6] Schrader C, Keussen C, Bewig B, von Freier A, Lins M (2005). Symptoms and signs of an acute myocardial ischemia caused by chemotherapy with Paclitaxel (Taxol) in a patient with metastatic ovarian carcinoma. Eur. J. Med. Res..

[7] Muggia F, Kudlowitz D (2014). Novel taxanes. Anticancer Drugs.

[8] Weng SF, Kai J, Guha IN, Qureshi N (2015). The value of aspartate aminotransferase and alanine aminotransferase in cardiovascular disease risk assessment. Open Heart..

[9] Li JH, Li TT, Wu XS, Zeng DL (2021). Effect of gamma globulin combined with creatine phosphate on viral myocarditis. Am. J. Transl. Res..

[10] Zhu T, Han Q, Zhang X, Hou Q (2021). Effects of Xinnaoning combined with trimetazidine on the levels of CK and its isoenzymes, AST, ALT and LDH in patients with myocardial ischemia. Am. J. Transl. Res..

[11] Boyer SW, Rajendiran S, Beaudin AE, Smith-Berdan S, Muthuswamy PK, Perez-Cunningham J (2019). Clonal and quantitative in vivo assessment of hematopoietic stem cell differentiation reveals strong erythroid potential of multipotent cells. Stem. Cell Rep..

[12] Wyss M, Kaddurah-Daouk R (2000). Creatine and creatinine metabolism. Physiol. Rev..

[13] Mehta RL, Cerdá J, Burdmann EA, Tonelli M, García-García G, Jha V (2015). International society of nephrology’s 0by25 initiative for acute kidney injury (zero preventable deaths by 2025): A human rights case for nephrology. Lancet.

[14] Udy AA, Scheinkestel C, Pilcher D, Bailey M (2016). Australian and New Zealand Intensive Care Society Centre for Outcomes and Resource Evaluation. The association between low admission peak plasma creatinine concentration and in-hospital mortality in patients admitted to intensive care in Australia and New Zealand. Crit. Care Med..

[15] Lillie JW, O'Keefe M, Valinski H, Hamlin HA, Varban ML, Kaddurah-Daouk R (1993). Cyclocreatine (1-carboxymethyl-2-iminoimidazolidine) inhibits growth of a broad spectrum of cancer cells derived from solid tumors. Cancer Res.

[16] Stocken DD, Hassan AB, Altman DG, Billingham LJ, Bramhall SR, Johnson PJ (2008). Modelling prognostic factors in advanced pancreatic cancer. Br. J. Cancer.

[17] Zhang DX, Dai YD, Yuan SX, Tao L (2012). Prognostic factors in patients with pancreatic cancer. Exp. Ther. Med..

[18] Valvona CJ, Fillmore HL, Nunn PB, Pilkington GJ (2016). The regulation and function of lactate dehydrogenase A: therapeutic potential in brain tumor. Brain Pathol..

[19] Yu SL, Xu LT, Qi Q, Geng YW, Chen H, Meng ZQ (2017). Serum lactate dehydrogenase predicts prognosis and correlates with systemic inflammatory response in patients with advanced pancreatic cancer after gemcitabine-based chemotherapy. Sci. Rep..

[20] Mishra D, Banerjee D (2019). Lactate dehydrogenases as metabolic links between tumor and stroma in the tumor microenvironment. Cancers (Basel)..

[21] Pérez-Tomás R, Pérez-Guillén I (2020). Lactate in the tumor microenvironment: An essential molecule in cancer progression and treatment. Cancers (Basel)..

[22] Semenza GL, Jiang BH, Leung SW, Passantino R, Concordet JP, Maire P (1996). Hypoxia response elements in the aldolase A, enolase 1, and lactate dehydrogenase A gene promoters contain essential binding sites for hypoxia-inducible factor 1. J. Biol. Chem..

[23] Urbańska K, Orzechowski A (2019). Unappreciated role of LDHA and LDHB to control apoptosis and autophagy in tumor cells. Int. J. Mol. Sci..

[24] Capula M, Mantini G, Funel N, Giovannetti E (2019). New avenues in pancreatic cancer: Exploiting microRNAs as predictive biomarkers and new approaches to target aberrant metabolism. Expert Rev. Clin. Pharmacol..

[25] Lv J, Zhou Z, Wang J (2019). Prognostic value of lactate dehydrogenase expression in different cancers: A meta-analysis. Am. J. Med. Sci..

[26] Lacourt TE, Kavelaars A, Tripathy D, Heijnen CJ (2022). Associations between fatigue and cellular metabolism in breast cancer patients: A longitudinal study. Psychoneuroendocrinology.

